# Management of polyethylene glycol solution aspiration using bronchoscopic lavage in a dog

**DOI:** 10.1111/jvim.15784

**Published:** 2020-05-07

**Authors:** Daniela M. Aguayo Santaella, Shelly J. Olin, Michael D. Willard, Jacqueline C. Whittemore

**Affiliations:** ^1^ Small Animal Clinical Sciences University of Tennessee College of Veterinary Medicine Knoxville Tennessee USA; ^2^ Department of Small Animal Clinical Sciences, TAMU‐4474 Texas A&M University College Station Texas USA

**Keywords:** acute respiratory distress syndrome, bronchoscopy, colonoscopy, endoscopic complication, GoLytely, pulmonary edema

## Abstract

Polyethylene glycol lavage solutions are used for colonic preparation in dogs and are considered relatively safe. Aspiration is an uncommon but potentially devastating complication of polyethylene glycol administration. Full recovery is possible and often rapid in people treated with bronchoalveolar lavage. A healthy 2‐year‐old male Beagle used in an endoscopy teaching laboratory aspirated a small amount of polyethylene glycol lavage solution. Although initially appearing unaffected, the dog quickly became hypoxemic. Bronchoscopy was used to lavage the lungs and aspirate tracheal/pulmonary fluid 5 times over the course of 45 minutes. The dog completely recovered. This report presents the successful treatment of polyethylene glycol aspiration in a dog. Although the seriousness of aspiration might not be immediately evident, bronchoscopy and lavage should be pursued because of the rapidly progressive nature of polyethylene glycol‐induced pulmonary edema.

**Abbreviations**
ARDS: acute respiratory distress syndrome
ICU: intensive care unit
PEG: polyethylene glycol

## 1 INTRODUCTION

Polyethylene glycol (PEG) commonly is used in dogs to prepare the colon for endoscopy.^1^ PEG polymers with high numbers of alternating hydrocarbon and oxygen repeats are used in colonic preparations because their large size prevents absorption.^4^ The most commonly reported adverse effects in dogs and people are nausea, vomiting, and bloating.^1^, ^2^ Aspiration of PEG‐containing solutions can cause pulmonary edema, acute respiratory distress syndrome (ARDS), multiple organ failure, and death.^1^, ^2^, ^3^ PEG aspiration results in edema and ARDS via 3 mechanisms: exudation of water from the pulmonary parenchyma into the airway, compromise of endogenous surfactant function and mucociliary clearance, and direct membranous damage.^4^, ^5^ Polyethylene glycol also can trigger local angioedema and anaphylaxis.^6^, ^7^ Treatment using bronchoscopic suction and airway lavage can result in full recovery. Although aspiration of PEG solutions appears to be uncommon in dogs, immediate treatment is imperative. This case report describes successful treatment of PEG aspiration in a dog.

## 2 CASE REPORT

A 2‐year‐old male castrated Beagle was evaluated for mild respiratory distress after aspiration of a PEG solution during colonic preparation for a gastrointestinal endoscopy in a continuing education laboratory. Earlier in the day, the dog had received docusate sodium (10 mg/kg PO once), maropitant (1 mg/kg IV once), warm water and medical lubricant enemas (20 mL/kg twice), and gavage via orogastric tube of 400 mL PEG solution (GoLytely, Braintree Laboratories Inc, Braintree, Massachusetts) twice without apparent incident. A third gavage of 400 mL PEG solution was performed via orogastric tube. All gavages were performed without sedation. Tube placement was confirmed for each gavage by auscultation and lack of cough during placement. During tube removal after the last gavage, the dog fell to his side and regurgitated a large volume of fluid onto the floor. At the end of the regurgitation episode, he gasped and aspirated a small amount of fluid. Based on visual comparison of the volume of fluid on the floor with instillation volume, the aspirated fluid was estimated to be 20 mL.

On physical examination performed immediately after aspiration, the dog was quiet with a slightly increased respiratory effort and an intermittent soft cough. Crackles could be ausculted in the right dorsal lung fields during coughing. Over the course of 5 minutes, the dog's respiratory effort gradually increased. The dog was premedicated with acepromazine (0.01 mg/kg IM), atropine (0.1 mg/kg IM), and butorphanol (0.4 mg/kg IM). An intravenous catheter was placed, and anesthesia was induced with propofol (3 mg/kg IV). Pulse oximetry was performed upon induction, 30 minutes after aspiration, revealing an SpO_2_ of 80%.

Bronchoscopy was performed using a 6.9 mm gastroscope (60511NKS, Karl Storz Veterinary Endoscopy, Goleta, California). Oxygen was administered using the flow‐by technique. Upon traversion of the arytenoids (Video S1), the trachea was found to be partially obstructed with clear foamy fluid. The fluid was removed using continuous, low‐pressure suction (<50 mm Hg) during advancement of the endoscope to the carina. The fluid completely filled RB1, with lesser degrees of filling of RB2 and RB4. Fluid filling of RB3 could not be accurately determined because of the filling of RB4. Fluid also was pulsing forward from LB1 and LB2, but it was not possible to determine the source because fluid from RB1 was observed pulsing craniad to the carina then caudally down the left mainstem bronchus. All visible fluid was meticulously suctioned from the airways, resulting in an increase in the dog's SpO_2_ to 100%. The trachea and bronchi then were lavaged with a balanced crystalloid solution (0.9% NaCL, 2 mL/kg), and the lavage fluid was removed via suction. The endoscope was removed to allow intubation of the dog, after which anesthesia was maintained using isoflurane in 100% oxygen.

The endoscope was reintroduced through an endotracheal bronchoscopic adaptor. Fluid was identified refilling multiple bronchi upon repeat bronchoscopy, and low‐pressure suction was repeated until all airways were apparently dry. The dog's body then was placed in the Trendelenburg position (supine with the dog's head positioned 30° below the head), resulting in rapid fluid egress from the lower airways, which filled the trachea and overflowed into the anesthetic circuit (Video S2). The airways were suctioned with the dog repositioned in the Trendelenburg position every 10 minutes until they remained free of fluid reaccumulation for more than 10 minutes. Between evaluations, the bronchoscope was withdrawn to the level of the bronchoscopic adapter to limit air trapping. The anesthetic circuit was disconnected, as needed, to remove fluid backflow from the trachea. Total procedure time was 45 minutes. Fluid collected in towels was not quantified. However, there was 220 mL of fluid collected in the suction canister (which included 20 mL of lavage fluid), 35 mL fluid in the anesthetic circuit, and approximately 35 mL fluid on the floor from prior clearance of the anesthetic circuit. No gastric contents were identified on gastroscopy.

The dog's vital signs remained stable throughout the procedure. Heart rate ranged from 75 to 105 bpm, respiratory rate from 35 to 47 bpm, systolic blood pressure from 90 to 110 mm Hg, and SpO_2_ remained ≥99%. Ten minutes after discontinuation of anesthesia, the dog was extubated. At time of extubation, its SpO_2_ was 100% with a respiratory rate of 30 bpm. The dog had a smooth recovery from anesthesia and, 15 minutes after extubation, was transported from the laboratory facility to the intensive care unit (ICU). At the time of transport, the dog had an SpO_2_ of 97% with a respiratory rate of 30 bpm.

Upon admission to the ICU 15 minutes later, the dog's SpO_2_ had decreased to 84% with a respiratory rate of 50 bpm. Terbutaline (0.01 mg/kg SQ) was administered to decrease potential PEG‐induced bronchospasm, and the dog was placed in an oxygen cage with a FiO_2_ of 40%. The dog's SpO_2_ increased to 99%, and his respiratory rate gradually decreased to 32 bpm. Over the course of the next 8 hours, the dog's SpO_2_ declined to 94%, and its respiratory rate increased to 52 bpm. Consideration was given to performing repeat bronchoscopic lavage, but the dog's respiratory effort remained mild. A second dose of terbutaline (0.01 mg/kg SQ) was administered. The dog became more comfortable and went to sleep. On repeat examination 3 hours later, the dog's SpO_2_ had increased to 99%. The only abnormality on physical examination was mild tachypnea, so the dog was weaned to room air approximately 24 hours after the aspiration event. Because of complete resolution of respiratory signs within 48 hours of the event, the dog was released to the colony and has remained clinically healthy with no abnormalities for 15 months since the aspiration event. No abnormalities were identified on thoracic radiographs taken 12 months after aspiration.

## 3 DISCUSSION

Polyethylene glycol aspiration previously has been reported to be fatal in dogs. This report describes the use of bronchoscopic alveolar lavage and repeated airway fluid suctioning to stabilize a dog that developed respiratory distress after regurgitating and aspirating PEG. The dog fully recovered within 48 hours.

Review of the literature revealed 21 human cases of PEG aspiration: 14 survivors,^2^, ^3^, ^8^, ^9^, ^10^, ^11^, ^12^, ^13^, ^14^ 6 fatalities,^4^, ^8^, ^9^, ^15^, ^16^, ^17^, ^18^ and 1 case of unknown outcome.^19^ Of the 14 survivors, 10 required mechanical ventilation because of rapid onset ARDS. Case management, reported for 7 cases, included glucocorticoid administration (5/7), airway suctioning (3/7), and lavage (4/7). Time to extubation was reported for 9 cases: <3 (7/9), 12 (1), and 21 days (1). The person requiring ventilation for 12 days had unchanged respiratory status for 10 days, with rapid improvement in respiratory status after therapeutic bronchoalveolar lavage was performed. The person requiring ventilation for 21 days underwent suction, but airway lavage was never performed.

All 6 deaths received immediate aggressive respiratory support, including mechanical ventilation. Treatment included glucocorticoids (2/6), airway suctioning (2/6), and unsuccessful lavage (1/6). Time to death was <3 (4/6), 14 (1/6), and 26 days (1/6). Five people died from ARDS, whereas 1 person died of severe stroke induced by prolonged hypoxemia.

Details regarding potential risk factors for aspiration were provided for 17 cases. Fourteen people received PEG by nasogastric or orogastric feeding tube,^2^, ^3^, ^4^, ^8^, ^9^, ^11^, ^12^, ^14^, ^15^, ^16^, ^18^, ^19^ whereas PEG was administered PO in 3 cases.^10^, ^13^, ^17^ Radiographic confirmation of correct tubing positioning was not performed before PEG administration in any case. Difficult placement was reported in 2 people, 2 tubes moved or were dislodged after placement, and 4 tubes were confirmed to be inappropriately positioned after clinical signs developed. Eight people vomited during or after PEG infusion. Other possible predisposing factors included underlying pulmonary disease (3), receipt of a drug with sedative properties (2), and “brutal cough,” concurrent hiatal hernia, or grand mal seizures (1 each).

Data on PEG aspiration in the veterinary literature is limited to 1 report of fatal PEG aspiration by an 8‐year‐old German shepherd dog. The dog received acepromazine before PEG administration via orogastric tube due to aggression.^1^ Details about attempted case management were not provided, but potential risk factors predisposing to aspiration included the use of sedation, which could have blunted the gag reflex, and administration of PEG by gavage. The majority of people suffering PEG aspiration were administered it via feeding tube. Thus, perhaps the simplest way to decrease PEG aspiration is by encouraging voluntary consumption. In our experience, many dogs will drink multiple dosages (30‐40 mL/kg) of PEG reconstituted using chicken broth, instead of water.

For cases that refuse PEG, it can be administered by either nasogastric or orogastric feeding tube. Positioning of nasogastric tubes should be confirmed via radiographs, preferably with contrast, before initial usage. For animals receiving repeated PEG dosages via nasogastric tube, auscultation of gastric sounds during administration of air boluses should be used to rule out tube migration before each use. Bedside nasogastric placement tests have been developed to screen for tube malpositioning in people. The devices detect carbon dioxide by siphoning air from the tube through an indicator paper using a bellows. These are used as an initial test of tube placement, with radiographs performed thereafter, and to confirm lack of tube migration between gavages. Because detection of malpositioning is dependent upon the amount of air pulled through the chamber, sensitivity is poor for tubes <8 Fr, limiting their use in small animals (personal observation, 2020). Correct positioning of orogastric tubes can be determined using palpation, auscultation of gastric sounds burbling up through the tube, or auscultation of the stomach while air is being infused through the tube.

To limit complications from PEG administration via gavage, premedication with an antiemetic is recommended.^20^ In 1 report of 46 dogs, regurgitation was less common for dogs administered maropitant (25% after first dose, 12.5% after second dose) 30 minutes before the first of 2 doses of PEG via orogastric tube, versus metoclopramide (52.4% after first dose, 31.6% after second dose).^20^ Although the difference was not statistically significant, type 2 error cannot be ruled based on the available data. Regurgitation was more common after the first PEG dosage even though antiemetic was not readministered. One possible explanation is that the dogs partially acclimated to rapid gastric filling. Alternatively, effects of the administered antiemetics might have continued to increase after administration of the first PEG dose. Further evaluation is warranted to determine the ideal timing of antiemetic pretreatment and assess whether use of an escalating PEG dosage protocol decreases the incidence of regurgitation.

Other factors associated with an increased likelihood of regurgitation after PEG administration include decreased dog size, female sex, and more rapid PEG administration, whereas differences in operator significantly affect the likelihood of vomiting.^20^ Based on these findings, highly experienced personnel should perform PEG gavages at a controlled rate in unsedated dogs after antiemetic administration, with use of a lower PEG dosage in smaller animals, and with dosage escalations based on animal tolerance.

In this case, the decision to perform bronchoscopy with lavage was made before the development of respiratory distress based on reports of PEG aspiration in people. It is possible that the dog would have made a full recovery if managed conservatively. However, this is considered unlikely given the rapid deterioration in respiratory status noted during anesthetic premedication, need for repeated airway suctioning during bronchoscopy, and final volume of fluid retrieved. Conversely, the use of a greater fluid volume, repeated lavage performance before extubation, or both might have prevented development of transient oxygen dependency. More aggressive lavage might be required to achieve a good outcome in animals with compromised mucociliary clearance. Supporting this possibility is the observation that only 1 of 3 people with compromised pulmonary function survived PEG aspiration.^4^, ^10^, ^15^ Fluid yield might have been improved had the dog been repositioned into dorsal recumbency before termination of bronchoscopy, a smaller diameter endoscope been used, or both. Finally, if the dog had been held aloft by the rear legs immediately after aspiration, this might have decreased respiratory exposure to PEG, thereby lessening the severity of the resultant pulmonary edema.

Although only a small volume of PEG was estimated to have been aspirated, a large volume of fluid was recovered from the dog's airways during bronchoscopic suction with apparent refilling of the airways between suction events. Veterinarians should be aware that the osmotic effect of PEG is marked, with immediate fluid exudation into the airways. Objective quantification of regurgitant volumes, in concert with suctioning of the stomach to quantitate residual gastric PEG volume, might aid in determining what percentage of airway fluid is from aspiration versus accumulation secondary to PEG's osmotic effects. Early, aggressive intervention in cases of PEG aspiration, potentially including bronchoalveolar lavage, can result in a positive outcome.

## CONFLICT OF INTEREST DECLARATION

Authors declare no conflict of interest.

## OFF‐LABEL ANTIMICROBIAL DECLARATION

Authors declare no off‐label use of antimicrobials.

## INSTITUTIONAL ANIMAL CARE AND USE COMMITTEE (IACUC) OR OTHER APPROVAL DECLARATION

The dog described in this report was on an IACUC protocol approved by the IACUC of the University of Tennessee, Knoxville (protocol number 1963) and performed in compliance with “The Guide for the Care and Use of Laboratory Animals” in laboratory animal facilities that are AAALAC certified.

## HUMAN ETHICS APPROVAL DECLARATION

Authors declare human ethics approval was not needed for this study.

## Supporting information


**Supplementary Video 1** Initial bronchoscopic evaluation performed 30 minutes after a dog aspirated a small volume of polyethylene glycol colonic lavage fluid. Note fluid filling multiple bronchi and welling cranially into the trachea. All visible fluid was meticulously suctioned from the airways, resulting in an increase in the dog's SpO_2_ to 100%.

**Supplementary Video 2** Bronchoscopic reevaluation performed 13 minutes after the airways had been suctioned until apparently dry twice. Rapid fluid egress is observed after the dog's body was lifted into the Trendelenburg position. The anesthetic circuit became obstructed with fluid, resulting in temporary discontinuation of bronchoscopy. The airways were suctioned with the dog in the Trendelenburg position every 10 minutes until they remained free of fluid reaccumulation.
